# Efficacy and Safety of Rituximab in Autoimmune Disease—Associated Interstitial Lung Disease: A Prospective Cohort Study

**DOI:** 10.3390/jcm11040927

**Published:** 2022-02-10

**Authors:** Natalia Mena-Vázquez, Rocío Redondo-Rodríguez, Marta Rojas-Gimenez, Carmen María Romero-Barco, Sara Manrique-Arija, Rafaela Ortega-Castro, Ana Hidalgo Conde, Rocío Arnedo Díez de los Ríos, Eva Cabrera César, Francisco Espildora, María Carmen Aguilar-Hurtado, Isabel Añón-Oñate, Lorena Pérez-Albaladejo, Manuel Abarca-Costalago, Inmaculada Ureña-Garnica, Maria Luisa Velloso-Feijoo, Maria Victoria Irigoyen-Oyarzábal, Antonio Fernández-Nebro

**Affiliations:** 1 Instituto de Investigación Biomédica de Málaga (IBIMA), 29010 Málaga, Spain; rocioredondo91@hotmail.com (R.R.-R.); menchu01@hotmail.com (C.M.R.-B.); sarama_82@hotmail.com (S.M.-A.); inuregar@gmail.com (I.U.-G.); maviirigoyen@gmail.com (M.V.I.-O.); afnebro@gmail.com (A.F.-N.); 2 UGC de Reumatología, Hospital Regional Universitario de Málaga, 29009 Málaga, Spain; 3 Departamento de Medicina, Universidad de Málaga, 29010 Málaga, Spain; 4 Instituto Maimónides de Investigación Biomédica de Córdoba (IMIBIC), 14004 Córdoba, Spain; rojasgimenezm@gmail.com (M.R.-G.); orcam84@hotmail.com (R.O.-C.); 5 UGC de Reumatología, Hospital Universitario Reina Sofía de Córdoba, 14004 Córdoba, Spain; 6 UGC de Reumatología, Hospital Clínico Universitario Virgen de la Victoria, 29010 Málaga, Spain; 7 Servicio de Medicina Interna, Hospital Universitario Virgen de la Victoria, 29009 Málaga, Spain; ahidalgoconde@gmail.com (A.H.C.); rocioardiez@gmail.com (R.A.D.d.l.R.); maabco@gmail.com (M.A.-C.); 8 UGC Neumología, Hospital Universitario Virgen de la Victoria, 29009 Málaga, Spain; evacabreracesar@gmail.com; 9 UGC de Neumología, Hospital Regional Universitario de Málaga, 29009 Málaga, Spain; fespildorahernandez@gmail.com; 10 UGC de Radiodiagnóstico, Hospital Regional Universitario de Málaga, 29009 Málaga, Spain; maguh007@gmail.com; 11 UGC de Reumatología, Hospital Universitario de Jaén, 23007 Jaén, Spain; isaanononate@gmail.com; 12 UGC de Reumatología, Hospital Universitario Virgen de las Nieves, 18014 Granada, Spain; lorenaperezalba@gmail.com; 13 UGC de Reumatología, Hospital Universitario Virgen de Valme, 41014 Sevilla, Spain; mlvelloso@hotmail.com

**Keywords:** autoimmune disease, interstitial lung disease, rituximab

## Abstract

Objectives: To analyze the efficacy and safety of rituximab (RTX) in connective tissue disease associated with interstitial lung disease (CTD-ILD). Methods: We performed a multicenter, prospective, observational study of patients with CTD-ILD receiving rituximab between 2015 and 2020. The patients were assessed using high-resolution computed tomography and pulmonary function tests at baseline, at 12 months, and at the end of follow-up. The main outcome measure at the end of follow-up was forced vital capacity (FVC) > 10% or diffusing capacity of the lungs for carbon monoxide (DLCO) > 15% and radiological progression or death. We recorded clinical characteristics, time to initiation of RTX, concomitant treatment, infections, and hospitalization. A Cox regression analysis was performed to identify factors associated with worsening ILD. Results: We included 37 patients with CTD-ILD treated with RTX for a median (IQR) of 38.2 (17.7–69.0) months. At the end of the follow-up, disease had improved or stabilized in 23 patients (62.1%) and worsened in seven (18.9%); seven patients (18.9%) died. No significant decline was observed in median FVC (72.2 vs. 70.8; *p* = 0.530) or DLCO (55.9 vs. 52.2; *p* = 0.100). The multivariate analysis showed the independent predictors for worsening of CTD-ILD to be baseline DLCO (OR (95% CI), 0.904 (0.8–0.9); *p* = 0.015), time to initiation of RTX (1.01 (1.001–1.02); *p* = 0.029), and mycophenolate (0.202 (0.04–0.8); *p* = 0.034). Only 28 of the 37 patients (75.6%) were still undergoing treatment with RTX: two patients (5.4%) stopped treatment due to adverse events and seven patients (18.9%) died owing to progression of ILD and superinfection. Conclusion: Lung function improved or stabilized in more than half of patients with CTD-ILD treated with RTX. Early treatment and combination with mycophenolate could reduce the risk of progression of ILD.

## 1. Introduction

Interstitial lung disease (ILD) is a common condition in patients with connective tissue disease (CTD). It is associated with increased morbidity and mortality [1]. The CTDs most commonly associated with ILD (CTD-ILDs) include systemic sclerosis (SS), rheumatoid arthritis (RA), and inflammatory myopathy (IM), which have been reported in up to 70% of affected patients [2]. ILD is the main cause of death in patients with SS and MI [3,4,5], while in RA patients, it is the second cause of death after cardiovascular disease [1]. While the treatment of these diseases has improved in recent years with the advent of immunosuppressants and biologics, management of ILD is clinically challenging, since patients are generally excluded from clinical trials for safety reasons [6].

Corticosteroids, cyclophosphamide, mycophenolate mofetil, and azathioprine are the most common drugs for CTD-ILD treatment [2]. While these immunosuppressants have proven beneficial for patients with CTD-ILD, the response proved to be insufficient in some cases, thus necessitating rescue therapy [7,8,9]. Furthermore, antifibrotic agents such as nintedanib were shown to be beneficial for lung involvement in patients from the SENSCIS [10] and INBUILD [11] studies. The use of conventional synthetic disease-modifying antirheumatic drugs (csDMARDs), generally in patients with RA and in some manifestations of SS and IM, has for some time been considered controversial in the treatment of CTD-ILD. Although older studies associated methotrexate with the development of ILD, more recent, higher-quality studies have failed to confirm the association [12,13]. Evidence is scarcer for the other csDMARDs. However, one meta-analysis did not report a higher frequency of respiratory adverse effects [14]. As for biologic DMARDS (bDMARDs), the available evidence—based mainly on cross-sectional and retrospective studies—suggests that rituximab and abatacept could be safe for CTD-ILD treatment [6,15,16,17,18,19,20,21,22]. Tocilizumab has also been suggested to be effective in preserving lung function in SS [23]. Tumor necrosis factor inhibitors, on the other hand, have been associated with a risk of exacerbating lung disease in patients with RA [14].

Rituximab is a chimeric monoclonal antibody that depletes anti-CD20 B cells and is composed of a human portion and a murine portion. It has been approved for the treatment of RA [24] and antineutrophil cytoplasmic antibody-associated vasculitis [25]. However, some recent retrospective studies suggest that it could be an alternative treatment for patients with CTD-ILD, even in cases that prove refractory to conventional immunosuppressants [6,21,26,27,28]. Therefore, based on a multicenter registry study of patients with CTD-ILD [21,22], we prospectively evaluated the use of rituximab with the following objectives: (1) to report on the efficacy and safety profile of rituximab in different CTD-ILDs; and (2) to identify risk factors that help to predict progression and mortality in patients treated with rituximab.

## 2. Materials and Methods

### 2.1. Design

We performed a multicenter prospective observational study of a cohort of patients with CTD-ILD receiving rituximab at 6 teaching hospitals in Andalusia, Spain. The study was approved by the Ethics Committee of Hospital Regional Universitario de Málaga (HRUM) (Code no. 1719-N-15). All the participants gave their written informed consent before participating. 

### 2.2. Study Population

Patients with CTD-ILD who were candidates for treatment with rituximab were recruited at the participating centers between March 2015 and June 2021. ILD was confirmed using pulmonary function testing (PFT) and high-resolution computed tomography (HRCT) or lung biopsy. The eligibility criteria were as follows: age ≥ 18 years; RA based on the criteria of ACR/EULAR 2010 [29]; SS based on the criteria of ACR/EULAR 2013 (41); and dermatomyositis and polymyositis (MI) based on the criteria of Bohan and Peter [30,31], as applicable; and treatment with ≥2 doses of rituximab for at least 12 months. We excluded patients with an inflammatory disease or rheumatic disease other than RA, SS, and IM (except for secondary Sjögren syndrome).

### 2.3. Protocol

All patients underwent a check-up every 3–6 months at the rheumatology clinic and every 6–12 months at the pulmonology clinic in cases requiring joint follow-up. Patients also underwent an HRCT scan and PFT at initiation of treatment with rituximab (V0) and, subsequently, at 12 months (V12) and when required, according to the criterion of the attending physician or because of worsening clinical condition. A final cut-off was made in 2020 with HRCT and PFT (Vf). All HRCT scans were based on axial slices (1.5 or 2.0 mm in thickness) taken at 1 cm intervals along the thorax and reconstructed using a high-spatial-frequency algorithm, with acquisition of 20–25 images per patient. The radiological evaluation was performed blind and independently by 2 experts in pulmonary radiology at HRUM. Discrepancies in the readings were resolved by consensus. Data were recorded at V0, V12, and during the last year of follow-up (Vf).

Patients who had worsening respiratory symptoms or decline in the pulmonary function tests compared to the time of ILD diagnosis were treated with rituximab. Rituximab was administered in 2 intravenous infusions of 1000 mg on days 1 and 15 every 6 months or more, depending on pulmonary or joint symptoms and serum immunoglobulin levels. All patients were premedicated at each infusion with 100 mg of methylprednisolone, antihistamines, and antipyretic agents. 

### 2.4. Working Definitions and Variables

The main variable was “Course of ILD at the end of follow-up (Vf)” in terms of improvement, stabilization, progression, or death. Improvement was defined as increased forced vital capacity (FVC) ≥ 10% or diffusing capacity of the lungs for carbon monoxide (DLCO) ≥ 15% and no radiological progression on the HRCT scan. Stabilization was defined as maintenance or increase in FVC ≤ 10% or DLCO < 15% and no radiological progression on the HRCT scan. Progression was defined as a decrease in FVC > 10% or DLCO > 15% and radiologic progression on the HRCT scan. Similarly, radiologic progression was considered an increase of ≥ 20% in the presence and extension of ground-glass opacities, reticulation, honeycombing, diminished attenuation, centrilobular nodules, other nodules, emphysema, and consolidation compared with the HRCT scan at V0. 

The ILD patterns were defined according to the lung biopsy or HRCT according to the standardized criteria of the American Thoracic Society/European Respiratory Society International Multidisciplinary Consensus Classification of the Idiopathic Interstitial Pneumonias [32] and classified as nonspecific interstitial pneumonia (NSIP), usual interstitial pneumonia (UIP), and other (bronchiolitis obliterans, organizing pneumonia, lymphoid interstitial pneumonia, and mixed). PFT comprised complete spirometry expressed as a percentage predicted and adjusted for age, sex, and height. A predicted FVC <80% was considered abnormal. DLCO was evaluated using the single-breath method corrected for hemoglobin (DLCO-SB) and was considered ab-normal when <80%. The conclusive diagnosis of CTD-ILD was formulated in a multidisciplinary context after excluding infections, drug toxicity, occupational exposure, smoking-related lung diseases, neoplasia, and emphysema.

Other variables included duration of symptoms, diagnostic delay, and smoking history (current or previous). We recorded infections, the hospitalization event, and causes of hospitalization. As for medication, we recorded csDMARDs, targeted synthetic DMARDs, bDMARDs, immunosuppressants, antifibrotic agents, and corticosteroids. We calculated the time from diagnosis of CTD-ILD until initiation of rituximab. We also collected laboratory values as follows: autoantibodies; rheumatoid factor (RF) (reference value (RV) 20 U/mL; high titer, > 60 U/mL), anticitrullinated peptide antibody (ACPA) (RV, 10 U/mL, high titer > 340 U/mL), antinuclear antibody (ANA), anti-U1RNP (MCTD), anti-Scl70, anti-RNA polymerase III, anti-PM-Scl (PM-Scl overlap), anti-Ro 52 kDa, anti-Ro 60 kDa, anti-La, anti-aminoacyl-tRNA synthetase, anti-Mi-2, anti-SRP, anti-TIF1, anti-NXP-2/MJ, anti-MDA5 (CADM), anti-HMGCR, and anti-SAE.

### 2.5. Statistical Analysis

We performed a descriptive analysis of the clinical, epidemiological, autoimmune, and therapy-related characteristics of all patients with CTD-ILD receiving rituximab. Qualitative variables were expressed as absolute numbers and percentages; quantitative variables were expressed as mean and standard deviation (SD) or median and interquartile range (IQR), depending on the normality of the distribution, as assessed using the Kolmogorov–Smirnov test. The χ^2^ test and ANOVA or Kruskal–Wallis test were used depending on normality to compare the main characteristics of the 3 groups of patients: (1) RA-ILD; (2) SS-ILD; and (3) IM-ILD. The bivariate analysis was performed using a paired *t* test or Wilcoxon test, as applicable, for V0-V12 and V0-Vf. We used Kaplan–Meier curves to estimate survival for patients with CTD-ILD receiving rituximab. Survival was measured from V0 until the end of follow-up (Vf) or death. Cox regression analysis was used to identify prognostic factors from time to progression or death using a univariate model and a multivariate model (forward stepwise). Dead patients without data for the terminal event were managed using the last-observation-carried-forward method. All variables that reached *p* < 0.10 were entered into the Cox multivariate model. Given an alpha risk of 0.05 and a beta risk of 0.2 in a bilateral contrast, the sample size calculation showed that 18 patients were necessary to detect an expected significant difference of FVC in 15.3 units and 30 patients were necessary to detect an expected significant difference in 7.2 units of DLCO for patients with CTD-ILD after 24 months of treatment with RTX [28]. We also analyzed the number of infections and hospitalization. The analysis was performed using the program R Commander. 

## 3. Results

### 3.1. Baseline Clinical Characteristics 

**Figure 1:**
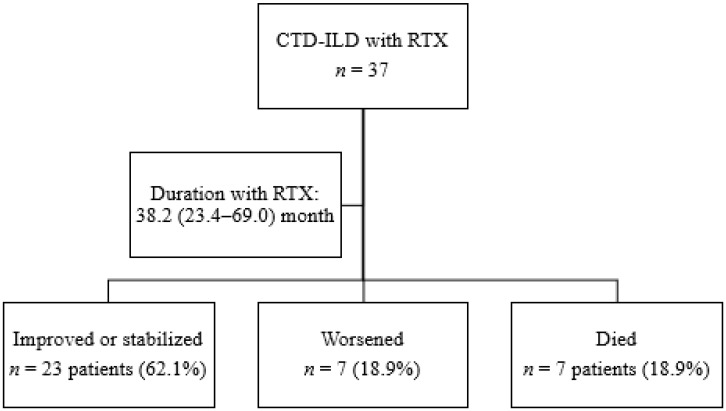
Flowchart showing the follow-up of patients.

A total of 37 patients with CTD-ILD were treated with rituximab for a median (IQR) of 38.2 (23.4–69.0) months. Figure 1 shows the progress of patients through the study. The main baseline characteristics are shown in Table 1. Of the 37 patients included, 19 had RA (51.4%), 14 had SS (37.8%), and 4 had IM (10.8%). No differences were detected between the three subgroups for duration of treatment with rituximab (*p* = 0.291). Mean age was 63 years, and more than half of the patients were women (73%). At initiation of rituximab, the median time from onset of ILD was 5.4 years. Almost half of the patients had been smokers or were active smokers at inclusion. More than 90% of patients with RA were RF- or ACPA-positive. The main findings in patients with SS were positive titers for anti-scl70 (50%) and anticentromere (21%); anti-PL-7 were the most frequent antibodies in patients with MI (50%).

At V0, 15 of the 37 patients (40.5%) were receiving a combination of rituximab and a csDMARD, 20 (54.1%) were receiving a combination of rituximab and an immunosuppressant, and 2 (5.4%) were receiving rituximab in monotherapy. Two patients with IM received rituximab combined with mycophenolate mofetil and hydroxychloroquine, and one patient with SS received rituximab combined with mycophenolate mofetil and methotrexate. More than half of the patients were taking corticosteroids. There were no differences between the subgroups of patients taking a combination with a csDMARD (*p* = 0.637). However, more patients with SA and IM were taking immunosuppressants combined with rituximab than those with RA (*p* = 0.044). The median (IQR) time from diagnosis of ILD to initiation of rituximab was 12.0 (6.5–48.2) months, with no differences between the subgroups (*p* = 0.455). 

Before V0, 23 patients (62%) had received at least 1 csDMARD, 11 (29%) had received a bDMARD, and 16 (43.2%) had received an immunosuppressant (Supplementary Table S1). The median number of previous csDMARDS was higher in patients with RA than in those with SS and MI (*p* < 0.001), as was the median number of previous bDMARDs (*p* = 0.001), whereas that of previous immunosuppressants was higher in patients with SS (*p* = 0.033).

Almost half of the patients had the UIP radiological pattern (48.6%), and almost half had the NSIP pattern (48.6%). Only one patient had a pattern compatible with fibrotic NSIP (2.7%). By patient subgroup, the NSIP pattern was predominant in SS (71.4%) and IM (100%), whereas the UIP pattern was more frequent in RA (73.7%). 

### 3.2. Clinical Course

Infection was recorded in 29 of the 37 patients (78.4%) during follow-up. These were mostly respiratory (70.3%), and almost half of the patients (43.2%) were hospitalized at least once. The most frequent reasons for hospitalization were respiratory infection (37.8%), followed by progression of ILD (27.0%). No significant differences were detected between the subgroups for infection (*p* = 0.985), hospitalization (*p* = 0.461), or mortality (*p* = 0.123) (Table 2). Seven patients died (18.9%): four owing to progression of ILD and superinfection, and three owing to progression of ILD. Supplementary Table S2 shows the duration of follow-up, the treatment administered, and the cause of death. 

In addition to the patients who died, only two patients discontinued rituximab permanently during follow-up: one (RA patient) owing to superinfected skin ulcers that proved refractory to antibiotics after 79 months with rituximab, and another (IM patient) owing to urinary tract infection and recurrent herpes simplex labialis after 24 months with rituximab.

### 3.3. Pulmonary Outcomes

**Figure 2:**
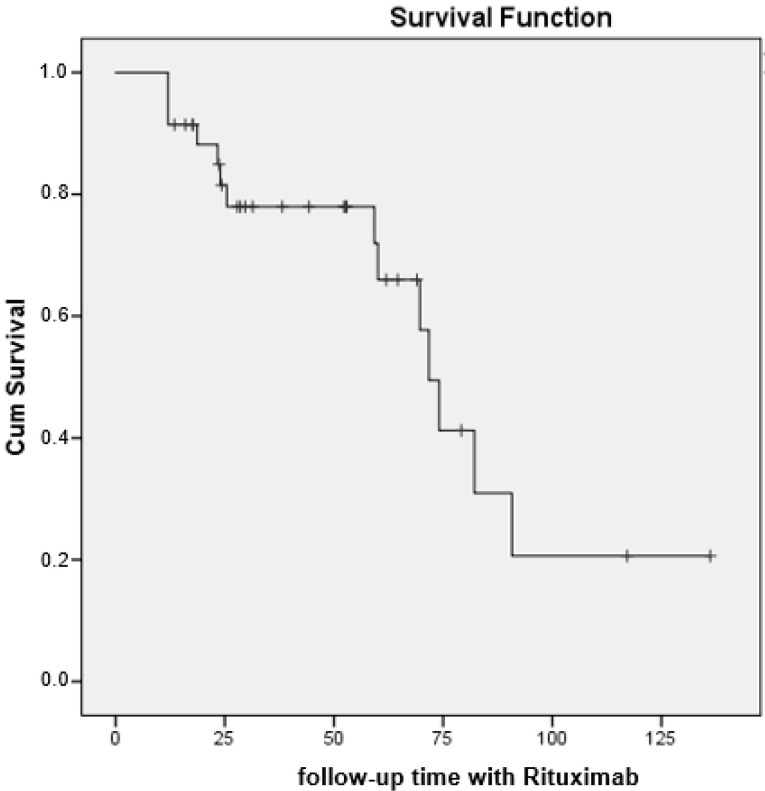
Survival curve (progression/mortality) in 37 patients with CTD-ILD receiving rituximab.

At the end of follow-up (see Table 3), the patients’ condition had improved or stabilized in more than half of the cases (62.2%) and had worsened or was fatal in 37.8% of cases (Table 2). There were no differences between the subgroups of patients treated with rituximab in terms of the primary endpoint of pulmonary outcome (*p* = 0.179). The median (95% CI) survival until progression of ILD or death was 71.8 months (65.2–78.3) (Figure 2).

**Figure 3:**
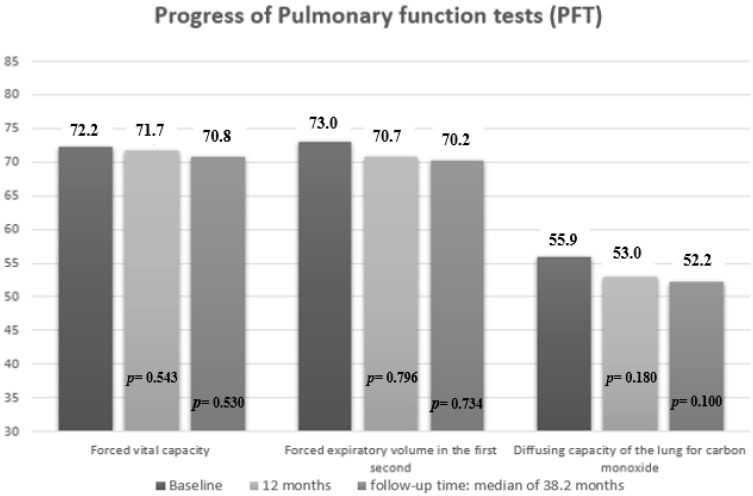
Pulmonary function results at 12 months and at the end of follow-up in patients with CTD-ILD receiving rituximab. P = *p* value for comparison between 12 months with baseline, and end of follow-up with baseline.

Mean PFT values decreased significantly at the start of RTX compared to the date of ILD diagnosis in FVC values (mean (SD), 72.2 (21.3) vs. 73.5 (16.9) mg/L; *p* = 0.040), DLCO-SB (mean (SD), 55.9 (15.7) vs. 58.3 (16.1) mg/L; *p* = 0.041) and FEV1 (mean (SD), 73.0 (18.8) vs. 76.1 (18.1) mg/L; *p* = 0.034) (Supplementary Table S3). On the other hand, mean PFT values did not decrease significantly during the first 12 months of treatment with rituximab compared with baseline or the end of follow-up (Figure 3). Similarly, by subgroup, no worsening was observed in the mean PFT values at 12 months or at the end of follow-up (Table 3).

HRCT revealed radiological progression in 14 of 37 patients (37.8%). Seven of 37 patients (18.9%) fulfilled the criteria for progression of ILD, and 7 of 37 patients (18.9%) died. HRCT revealed no differences between the subgroups with respect to progression (*p* = 0.142). Of the seven patients who died, six (31.6%) had RA and one (7.1%) had SS.

### 3.4. Factors Associated with Progression of ILD in Patients with CTD-ILD Treated with Rituximab

Supplementary Table S4 shows the results of the bivariate analysis between patients with CTD-ILD treated with rituximab with and without progression of ILD. Both groups were equivalent in terms of epidemiological, clinical, and radiological characteristics. However, as compared with patients whose condition improved/stabilized, those whose ILD progressed or who died from ILD had more chronic disease (median (IQR), 86.5 (51.9–130.8) vs. 54.9 (26.0–93.4) months; *p* = 0.046) and lower FVC values (mean (SD), 61.7 (14.7) vs. 75.5 (19.0) mg/L; *p* = 0.045) and DLCO-SB (mean (SD), 49.5 (13.3) vs. 59.8 (16.0) mg/L; *p* = 0.036) at onset of ILD. They also started treatment with rituximab later (median (IQR), 42.4 (14.2–84.6) vs. 7.4 (6.0–22.7) months; *p* = 0.016) and less frequently took the combination of mycophenolate mofetil and rituximab (*n* (%), 4 (28.6) vs. 15 (65.2) mg/L; *p* = 0.031).

Table 4 shows the results of the multivariate Cox analysis (DV: progression or death) in 37 patients with CTD-ILD for a median (IQR) time in treatment with rituximab of 38.2 (23.4–69.0) months. The event progression or mortality was recorded in 14 of the 37 patients. According to this model (Table 4), the best results were obtained in patients treated early and with better baseline values in the diffusion tests. The multivariate analysis identified that the combination of rituximab and mycophenolate mofetil, and the DLCO value were associated with reduced risk of progression of ILD in patients with CTD-ILD, whereas of delay in the initiation of rituximab after the diagnosis of ILD was associated with a higher probability of progression of lung disease (Table 4).

## 4. Discussion

We performed a prospective evaluation of lung function in 37 patients with CTD-ILD receiving treatment with rituximab and found that after a median of 38 weeks’ follow-up, ILD improved or stabilized in almost two-thirds of cases. Other studies have shown the beneficial effect of rituximab in CTD-ILD [6,21,26,27,28].

A series of multicenter clinical studies from EUSTAR showed that rituximab can stabilize and improve lung function in patients with SS-ILD [33] and in patients with antisynthetase syndrome [28]. However, other authors reached contrasting conclusions, especially with respect to the efficacy of rituximab in ILD associated with other CTDs, and particularly with RA-ILD, given that survival in affected patients has been shown to be lower than in other CTD-ILDs [34,35]. A recent study [15] showed that rituximab can be effective as a rescue therapy in a considerable percentage of patients with progressive RA-ILD that does not respond to standard treatment. In our study, these differences were not statistically significant, despite the higher number of patients whose disease progressed or who died among those with RA treated with rituximab. 

As for lung function evaluated using PFT, we found that disease had stabilized after 12 months in all the subgroups of CTD-ILD treated with rituximab and that it remained stable until the end of follow-up. These results agree with those of a recent meta-analysis, in which rituximab was superior to other immunosuppressants for the stabilization or improvement of FVC and DLCO in patients with CTD-ILD [28,36]. In our study, HRCT also revealed radiological stabilization/improvement in almost two-thirds of patients; this was the same for both the UIP and NSIP patterns. Consequently, rituximab might be able to curb progression of both patterns in a large percentage of patients with CTD-ILD. Similar findings have been reported elsewhere [6,37].

However, more than one-third of the patients in our study progressed poorly (ILD worsened in 18.9% and a similar percentage died). The factors associated with progression and mortality included poor baseline DLCO. In their cohort of patients with RA-ILD treated with rituximab for more than 10 years, Md Yusof et al. found that DLCO < 46% before initiation of rituximab was associated with progression of ILD. This finding points to the need for close follow-up of affected patients at initiation of rituximab. The authors recommend that if the patient’s condition continues to deteriorate, then alternative treatments, such as cyclophosphamide, antifibrotic agents, and lung transplant, should be considered [6].

Of note, we found that the combination of rituximab with mycophenolate mofetil reduced the risk of progression of ILD and death at the end of follow-up by 80%. Mycophenolate mofetil has proven effective for the treatment of ILD in patients with SS [38,39,40,41,42,43], although also in those with other CTDs [44,45]. Similarly, studies published in recent years show that combining rituximab with mycophenolate mofetil is well tolerated, safe, and potentially effective for the treatment of lung involvement in patients with SS-ILD [46,47]. It seems that the action of mycophenolate, mainly on T cells, and that of rituximab, on B cells, could exert an additive effect on the control of the immune response in affected patients. To this end, the ongoing clinical trial EvER-ILD (NCT02990286) aims to compare mycophenolate in monotherapy with the combination of mycophenolate and rituximab in patients with SS-ILD whose first-line immunosuppressants failed [48]. While awaiting the results of this trial, we can use data from observational studies to improve the therapeutic management of these patients.

In our study, the delay in initiating treatment with rituximab after diagnosis of ILD was more frequently associated with progression/mortality. These differences may arise because patients in whom initiation of rituximab was delayed are those whose previous immunosuppressive therapy failed and who therefore progressed more poorly. However, as shown in a recent meta-analysis, rituximab is more effective for improving or stabilizing FVC and has a better safety profile in patients with CTD-ILD than standard treatment with immunosuppressants. However, given its high cost, rituximab is not considered a replacement for standard treatment as the first option; therefore, rituximab may be a better option for patients who do not respond to standard treatment or who experience adverse effects [36]. 

As for the safety profile of rituximab, we found that infections were frequent and that, together with progression of ILD, were the cause of most deaths. However, only two patients discontinued rituximab permanently. Other studies in patients with CTD-ILD treated with rituximab recorded infections similar to those we report and found that these were the cause of most deaths [49,50]. However, the total number of infections and the mortality were similar to those of patients with CTD-ILD who did not receive rituximab [6]. The clinical course of ILD can be complicated by a variety of events, including infection caused by various respiratory pathogens, including bacteria, fungi, viruses, and mycobacteria. Despite the improvement in public health measures and antituberculous chemotherapy, pulmonary tuberculosis remains a common disease worldwide, particularly in developing countries. Latent tuberculosis can be reactivated and cause disease in patients under corticosteroid and/or immunosuppressive treatment. For this reason, before starting biological treatment, it is necessary to complete the active tuberculosis treatment [51].

Our study is subject to a series of limitations. First, the fact that the study was multicenter could lead to differences in the evaluation of lung function. We mitigated this limitation using centralized HRCT, since the radiological results were available online. Furthermore, the prospective design ensured low frequencies of missing data. Second, basing the study on various CTDs with different pathogenic mechanisms, disease duration, and associated treatment made for a more heterogeneous sample, thus hampering the identification of the effect of treatment and predictors of response. However, our prospective evaluation of these same subgroups enabled us to determine not only the progress of all the CTD-ILDs included, but also that of each group individually. On the other hand, the comedication (DMARDs) differed between diseases and was not controlled by study design. This is due to the fact that, in clinical practice, patients with RA have a predominance of joint involvement, which means that these patients receive more DMARDs than the other groups. However, we were able to fulfill the main objective of the study, which was to report on the efficacy and safety profile of rituximab in different CTD-ILDs, showing the results in each of them separately. Lastly, despite there being no differences between the subgroups of patients treated with rituximab in terms of the primary endpoint of pulmonary outcome (*p* = 0.179), there was a greater number of patients with SS who presented progression compared to RA and IM [15,52]. However, the low number of cases included may not be sufficient to reveal significant differences between the groups, despite most studies to date being based on small retrospective observational studies. Clinical trials with larger patient samples are necessary to generate more evidence. 

## 5. Conclusions

In conclusion, we found that lung function stabilized or improved after a median of 38 months of follow-up in more than half of patients with CTD-ILD receiving rituximab. No increases in the frequency of infection were recorded. Combination with mycophenolate could reduce the risk of progression of ILD and death by 80%. The delay in initiating treatment with rituximab and lower DLCO values were the main factors associated with progression of ILD and death. Therefore, patients should be followed closely, and other types of treatment (e.g., cyclophosphamide, antifibrotic agents, and lung transplant) should be considered.

## Figures and Tables

**Table 1:** Baseline demographic and clinical characteristics of 37 patients with CTD-ILD receiving rituximab.

Variable	Total *n* = 37	RA *n* = 19	SS *n* = 14	IM *n* = 4	*p* Value
Epidemiological characteristics					
Female sex, *n* (%)	27 (73.0)	13 (68.4)	11 (78.6)	3 (75.0)	0.806
Age in years, mean (SD)	62.8 (9.9)	67.7 (9.7)	57.9 (7.9)	56.6 (5.5)	0.001
Caucasian race, *n* (%)	36 (97.3)	19 (100.0)	13 (92.9)	4 (100.0)	0.430
Clinical–analytical characteristics					
Smoking					0.147
Never smoked, *n* (%)	20 (54.1)	9 (47.4)	7 (50.0)	4 (100.0)	
Smoked at some stage, *n* (%)	17 (45.9)	10 (52.6)	7 (50.0)	0 (0.0)	
Duration of CTD, months, median (IQR)	107.8 (49.5–188.8)	151.0 (8.0–240.5)	89.6 (51.3–184.4)	35.1 (25.1–49.0)	0.017
Duration of ILD, months, median (IQR)	65.4 (31.1–110.3)	82.2 (37.4–120.1)	64.5 (35.5–107.1)	25.9 (25.0–36.0)	0.136
RF-positive (>10) *n* (%)	19 (51.4)	19 (100.0)	0 (0.0)	0 (0.0)	<0.001
ACPA (<20), *n* (%)	18 (48.6)	18 (94.7)	0 (0.0)	0 (0.0)	<0.001
ANA-positive, *n* (%)	24 (64.9)	6 (31.6)	14 (100.0)	4 (100.0)	<0.001
Anti-scl70, *n* (%)	7 (18.9)	0 (0.0)	7 (50.0)	0 (0.0)	<0.001
Anticentromere, *n* (%)	3 (9.0)	0 (0,0)	3 (21,4)	0 (0,0)	0.156
Anti-RNA polymerase 3, *n* (%)	1 (2.7)	0 (0.0)	1 (7.1)	0 (0.0)	0.430
Anti Ku, *n* (%)	1 (2.7)	0 (0.0)	1 (7.1)	0 (0.0)	0.430
Anti-PL7, *n* (%)	2 (5.4)	0 (0.0)	0 (0.0)	2 (50.0)	<0.001
Anti-EJ, *n* (%)	1 (2.7)	0 (0.0)	0 (0.0)	1 (25.0)	0.014
Anti-TIF, *n* (%)	1 (2.7)	0 (0.0)	0 (0.0)	1 (25.0)	0.014
Treatment					
Time to initiation of rituximab *, median (IRQ)	12.0 (6.5–48.2)	25.1 (7.0–57.6)	11.4 (3.9–43.6)	7.4 (7.0–10.4)	0.455
Duration of treatment with rituximab, median (IQR) **	38.2 (23.4–69.9)	45.3 (22.2–79.9)	52.5 (24.7–63.3)	22.8 (17.7–36.2)	0.291
Combined with csDMARDs, *n* (%)	15 (40.5)	9 (47.4)	5 (35.7)	1 (25.0)	0.637
Methotrexate, *n* (%)	5 (13.5)	2 (10.5)	3 (21.4)	0 (0.0)	0.468
Leflunomide, *n* (%)	2 (5.4)	2 (10.5)	0 (0.0)	0 (0.0)	0.367
Sulfasalazine, *n* (%)	1 (2.7)	1 (5.3)	0 (0.0)	0 (0.0)	0.615
Hydroxychloroquine, *n* (%)	7 (18.9)	4 (21.1)	2 (14.3)	1 (25.0)	0.840
Combination with immunosuppressants, *n* (%)	20 (54.1)	7 (36.8)	9 (64.3)	4 (100.0)	0.044
Mycophenolate, *n* (%)	19 (51.4)	6 (31.6)	9 (64.3)	4 (100.0)	0.021
Azathioprine, *n* (%)	1 (2.7)	1 (5.3)	0 (0.0)	0 (0.0)	0.615
Corticosteroids, *n* (%)	25 (67.6)	14 (73.7)	7 (50.0)	4 (100.0)	0.121
Doses of corticosteroids, median (IQR)	5.0 (0.0–10.0)	5.0 (0.0–10.0)	2.5 (0.0–7.5)	10.0 (8.1–10.5)	0.519

Abbreviation: CTD: connective tissue disease; ILD: interstitial lung disease; RTX: rituximab; RA: rheumatoid arthritis; IM: inflammatory myopathy; SS: systemic sclerosis; RF: rheumatoid factor; ACPA: anticitrullinated peptide antibodies; ANA: antinuclear antibody; csDMARD: conventional synthetic disease-modifying antirheumatic drug; SD: standard deviation; IQR: interquartile range; * Time from diagnosis of ILD to initiation of rituximab ** Time from initiation of treatment with rituximab to end of follow-up or mortality. Statistical tests used: Pearson chi-squared (χ^2^), ANOVA, and Kruskal–Wallis.

**Table 2:** Clinical events in 37 patients with CTD-ILD receiving rituximab.

Variable	Total *n* = 37	RA *n* = 19	SS *n* = 14	IM *n* = 4	*p* Value
Infections, *n* (%)	29 (78.4)	15 (78.9)	11 (78.6)	3 (75.0)	0.985
Respiratory infection, *n* (%)	26 (70.3)	13 (68.4)	10 (71.4)	3 (75.0)	0.959
Other infections, *n* (%)	10 (27.0)	5 (26.3)	4 (28.6)	1(25.0)	0.980
Herpes simplex labialis, *n* (%)	2 (5.4)	1 (5.2)	0 (0.0)	1 (25.0)	0.333
Cutaneous involvement, *n* (%)	5 (13.5)	2 (10.5)	3 (21.4)	0 (0.0)	0.401
Urinary tract infection, *n* (%)	5 (13.5)	3 (15.7)	1 (7.1)	1 (25.0)	0.560
Hospitalization, *n* (%)	16 (43.2)	10 (52.6)	5 (35.7)	1 (25.0)	0.461
Reasons for hospitalization					0.360
Progression of ILD, *n* (%)	10 (27.0)	7 (36.8)	3 (21.4)	0 (0.0)	
Respiratory infection, *n* (%)	14 (37.8)	7 (36.8)	6 (42.8)	1 (25.0)	
Mortality, *n* (%)	7 (18.9)	6 (31.6)	1 (7.1)	0 (0.0)	0.123

Abbreviations: CTD: connective tissue disease; ILD: interstitial lung disease; RA: rheumatoid arthritis; MI: inflammatory myopathy; SS: systemic sclerosis. Statistical tests used: Pearson chi-squared (χ^2^), ANOVA, and Kruskal–Wallis.

**Table 3:** Results of pulmonary function testing in 37 patients with CTD-ILD receiving rituximab.

Variable		Total *n* = 37	RA *n* = 19	SS *n* = 14	IM *n* = 4	*p* Value
Outcome *						0.179
Improvement, *n* (%)	Final	6 (16.2)	1 (5.3)	4 (28.6)	1 (25.0)	
Stabilization, *n* (%)	Final	17 (45.9)	9 (47.4)	5 (35.7)	3 (75.0)	
Worsening, *n* (%)	Final	7 (18.9)	3 (15.8)	4 (28.6)	0 (0.0)	
Death, *n* (%)	Final	7 (18.9)	6 (31.6)	1 (7.1)	0 (0.0)	
Pulmonary function tests						
FVC, mean (SD)	Baseline	72.2 (21.3)	69.1 (15.0)	71.6(21.7)	79.0 (15.0)	0.644
	Final	70.8 (18.6)	67.4 (20.2)	70.7(25.2)	81.5 (10.0)	0.312
FVC < 80%, *n* (%)	Baseline	24 (64.9)	12 (63.2)	11 (78.6)	1 (25.0)	0.138
	Final	25(67.6)	15 (78.9)	9 (64.3)	1 (25.0)	0.105
FEV_1_, mean (SD)	Baseline	73.0 (18.8)	69.8 (16.0)	76.9 (24.9)	76.2 (7.5)	0.570
	Final	70.2 (18.7)	67.1 (19.9)	72.7 (19.7)	78.0 (8.0)	0.516
DLCO-SB, mean (SD)	Baseline	55.9 (15.7)	56.2 (17.7)	52.8 (15.6)	58.0 (5.0)	0.935
	Final	52.2 (17.0)	53.8 (19.4)	48.3 (15.2)	57.1 (4.0)	0.577
HRCT pattern						
Radiologic type						0.011
UIP, *n* (%)	Baseline	18 (48.6)	14 (73.7)	4 (28.6)	0 (0.0)	
	Final	18 (48.6)	14 (73.7)	4 (28.6)	0 (0.0)	
NSIP, *n* (%)	Baseline	18 (48.6)	4 (21.1)	10 (71.4)	4 (100.0)	
	Final	18 (48.6)	4 (21.1)	10 (71.4)	4 (100.0)	
Fibrotic NSIP, *n* (%)	Baseline	1 (2.7)	1 (5.3)	0 (0.0)	0 (0.0)	
	Final	1 (2.7)	1 (5.3)	0 (0.0)	0 (0.0)	
Progress on HRCT						0.142
Progression, *n* (%)	Final	14 (37.8)	9 (47.4)	5 (35.7)	0 (0.0)	
Stabilization, *n* (%)	Final	16 (43.2)	9 (47.4)	5 (35.7)	2 (50.0)	
Improvement, *n* (%)	Final	7 (18.9)	1 (5.3)	4 (28.6)	2 (50.0)	

Abbreviations: CTD: connective tissue disease; ILD: interstitial lung disease; RA: rheumatoid arthritis; IM: inflammatory myopathy; SS: systemic sclerosis; FVC: forced vital capacity; FEV1: forced expiratory volume in the first second; DLCO: diffusing capacity of the lungs for carbon dioxide; UIP: usual interstitial pneumonia; NSIP: nonspecific interstitial pneumonia; HRCT: high-resolution computed tomography; * Total progression of ILD: taking into account HRCT and pulmonary function testing (FVC and DLCO). Statistical tests used: Pearson chi-squared (χ^2^), ANOVA, Kruskal–Wallis, paired *t* test, and Wilcoxon test.

**Table 4:** Results of the multivariate analysis of progression of lung disease or mortality in patients with CTD-ILD receiving rituximab. Cox regression model (adjusted for time of treatment with rituximab).

Variable	Univariate HR (95% CI)	Multivariate HR (95% CI)	*p* Value
Age in years	1.007 (0.94–1.06)		
Sex, male	0.756 (0.21–2.72)		
Current or previous history of smoking	2.074 (0.77–6.04)		
Radiological pattern, UIP	1.200 (0.38–3.73)		
Progression of ILD, months	1.001 (0.99–1.01)		
Baseline FVC	0.956 (0.92–0.99)		
Baseline DLCO-SB	0.949 (0.91–0.98)	0.904 (0.83–0.98)	0.015
Time to initiation of rituximab, months	1.010 (1.00–1.01)	1.011 (1.00–1.02)	0.029
csDMARDs	0.877 (0.29–2.57)		
Combination with mycophenolate	0.252 (0.06–0.92)	0.202 (0.04–0.88)	0.034
Corticosteroids	0.667 (0.21–2.12)		

Abbreviations. CTD: connective tissue disease; ILD: interstitial lung disease; UIP: usual interstitial pneumonia; FVC: forced vital capacity; DLCO-SB: diffusing capacity of the lung for carbon monoxide, single-breath method; csDMARDs (methotrexate, leflunomide, hydroxychloroquine, sulfasalazine): Independent variables included in the equation: sex, age, baseline FVC, baseline DLCO-SB, time to initiation of rituximab, mycophenolate.

## Data Availability

Data presented in this study are available on request from the corresponding author.

## Supplementary Materials

The following supporting information can be downloaded at: https://www.mdpi.com/article/10.3390/jcm11040927/s1. Table S1: Previous treatment in 37 patients with CTD-ILD receiving rituximab. Table S2: Characteristics of patients with CTD-ILD receiving treatment with rituximab who died. Table S3: Pulmonary function results at ILD diagnosis and at baseline in patients with CTD-ILD. Table S4: Factors associated with pulmonary outcomes in patients with CTD-ILD receiving rituximab.

## References

[1] Robles-Pérez A., Luburich P., Bolivar S., Dorca J., Nolla J.M., Molina-Molina M., Narváez J.A. (2020). A prospective study of lung disease in a cohort of early rheumatoid arthritis patients. Sci. Rep..

[2] Demoruelle M.K., Mittoo S., Solomon J.J. (2016). Connective tissue disease-related interstitial lung disease. Best Pract. Res. Clin. Rheumatol..

[3] Steen V.D., Medsger T.A. (2007). Changes in causes of death in systemic sclerosis, 1972–2002. Ann. Rheum. Dis..

[4] Cottin V., Thivolet-Béjui F., Reynaud-Gaubert M., Cadranel J., Delaval P., Ternamian P.J., Cordier J.F. (2003). Interstitial lung disease in amyopathic dermatomyositis, dermatomyositis and polymyositis. Eur. Respir. J..

[5] Chan C., Ryerson C.J., Dunne J.V., Wilcox P.G. (2019). Demographic and clinical predictors of progression and mortality in connective tissue disease-associated interstitial lung disease: A retrospective cohort study. BMC Pulm. Med..

[6] Md Yusof M.Y., Kabia A., Darby M., Lettieri G., Beirne P., Vital E.M., Dass S., Emery P. (2017). Effect of rituximab on the progression of rheumatoid arthritis-related interstitial lung disease: 10 years’ experience at a single centre. Rheumatology (Oxford).

[7] Saketkoo L.A., Espinoza L.R. (2008). Rheumatoid arthritis interstitial lung disease: Mycophenolate mofetil as an antifibrotic and disease-modifying antirheumatic drug. Arch. Intern. Med..

[8] Oldham J.M., Lee C., Valenzi E., Witt L.J., Adegunsoye A., Hsu S., Chen L., Montner S., Chung J.H., Noth I. (2016). Azathioprine response in patients with fibrotic connective tissue disease-associated interstitial lung disease. Respir. Med..

[9] Barnes H., Holland A.E., Westall G.P., Goh N.S., Glaspole I.N. (2018). Cyclophosphamide for connective tissue disease-associated interstitial lung disease. Cochrane Database Syst. Rev..

[10] Distler O., Brown K.K., Distler J.H.W., Assassi S., Maher T.M., Cottin V., Varga J., Coeck C., Gahlemann M., Sauter W. (2017). Design of a randomised, placebo-controlled clinical trial of nintedanib in patients with systemic sclerosis-associated interstitial lung disease (SENSCIS™). Clin. Exp. Rheumatol..

[11] Flaherty K.R., Wells A.U., Cottin V., Devaraj A., Walsh S.L.F., Inoue Y., Richeldi L., Kolb M., Tetzlaff K., Stowasser S. (2019). Nintedanib in Progressive Fibrosing Interstitial Lung Diseases. N. Engl. J. Med..

[12] Ibfelt E.H., Jacobsen R.K., Kopp T.I., Cordtz R.L., Jakobsen A.S., Seersholm N., Shaker S.B., Dreyer L. (2020). Methotrexate and risk of interstitial lung disease and respiratory failure in rheumatoid arthritis: A nationwide population-based study. Rheumatology (Oxford).

[13] Kiely P., Busby A.D., Nikiphorou E., Sullivan K., Walsh D.A., Creamer P., Dixey J., Young A. (2019). Is incident rheumatoid arthritis interstitial lung disease associated with methotrexate treatment? Results from a multivariate analysis in the ERAS and ERAN inception cohorts. BMJ Open.

[14] Cubero C.C., Carmona E.C., Casasempere P.V. (2020). Systematic review of the impact of drugs on diffuse interstitial lung disease associated with rheumatoid arthritis. Reumatol. Clín..

[15] Narváez J., Robles-Pérez A., Molina-Molina M., Vicens-Zygmunt V., Luburich P., Yañez M.A., Alegre J.J., Nolla J.M. (2020). Real-world clinical effectiveness of rituximab rescue therapy in patients with progressive rheumatoid arthritis-related interstitial lung disease. Semin. Arthritis Rheum..

[16] Fernández-Díaz C., Loricera J., Castañeda S., López-Mejías R., Ojeda-García C., Olivé A., Rodríguez-Muguruza S., Carreira P.E., Pérez-Sandoval T., Retuerto M. (2018). Abatacept in patients with rheumatoid arthritis and interstitial lung disease: A national multicenter study of 63 patients. Semin. Arthritis Rheum..

[17] Fernández-Díaz C., Castañeda S., Melero-González R.B., Ortiz-Sanjuán F., Juan-Mas A., Carrasco-Cubero C., Casafont-Solé I., Olivé A., Rodríguez-Muguruza S., Almodóvar-González R. (2020). Abatacept in interstitial lung disease associated with rheumatoid arthritis: National multicenter study of 263 patients. Rheumatology (Oxford).

[18] Manfredi A., Cassone G., Furini F., Gremese E., Venerito V., Atzeni F., Arrigoni E., Della Casa G., Cerri S., Govoni M. (2020). Tocilizumab therapy in rheumatoid arthritis with interstitial lung disease: A multicenter retrospective study. Intern. Med. J..

[19] Bosello S.L., De Luca G., Rucco M., Berardi G., Falcione M., Danza F.M., Pirronti T., Ferraccioli G. (2015). Long-term efficacy of B cell depletion therapy on lung and skin involvement in diffuse systemic sclerosis. Semin. Arthritis Rheum..

[20] Marie I., Dominique S., Janvresse A., Levesque H., Menard J.F. (2012). Rituximab therapy for refractory interstitial lung disease related to antisynthetase syndrome. Respir. Med..

[21] Mena-Vázquez N., Godoy-Navarrete F.J., Manrique-Arija S., Aguilar-Hurtado M.C., Romero-Barco C.M., Ureña-Garnica I., Espildora F., Añón-Oñate I., Pérez-Albaladejo L., Gomez-Cano C. (2021). Non-anti-TNF biologic agents are associated with slower worsening of interstitial lung disease secondary to rheumatoid arthritis. Clin. Rheumatol..

[22] Mena-Vázquez N., Rojas-Gimenez M., Romero-Barco C.M., Manrique-Arija S., Francisco E., Aguilar-Hurtado M.C., Añón-Oñate I., Pérez-Albaladejo L., Ortega-Castro R., Godoy-Navarrete F.J. (2021). Predictors of Progression and Mortality in Patients with Prevalent Rheumatoid Arthritis and Interstitial Lung Disease: A Prospective Cohort Study. J. Clin. Med..

[23] Khanna D., Lin C.J.F., Furst D.E., Wagner B., Zucchetto M., Raghu G., Martinez F.J., Goldin J., Siegel J., Denton C.P. (2021). Long-Term Safety and Efficacy of Tocilizumab in Early Systemic Sclerosis-Interstitial Lung Disease: Open Label Extension of a Phase 3 Randomized Controlled *Trial. Am. J. Respir. Crit. Care Med.*
**2021**. Am. J. Respir. Crit. Care Med..

[24] Smolen J.S., Landewé R., Bijlsma J., Burmester G., Chatzidionysiou K., Dougados M., Nam J., Ramiro S., Voshaar M., van Vollenhoven R. (2017). EULAR recommendations for the management of rheumatoid arthritis with synthetic and biological disease-modifying antirheumatic drugs: 2016 update. Ann. Rheum. Dis..

[25] Yates M., Watts R.A., Bajema I.M., Cid M.C., Crestani B., Hauser T., Hellmich B., Holle J.U., Laudien M., Little M.A. (2016). EULAR/ERA-EDTA recommendations for the management of ANCA-associated vasculitis. Ann. Rheum. Dis..

[26] Matteson E.L., Bongartz T., Ryu J.H., Crowson C.S., Hartman T.E., Dellaripa P.F. (2012). Open-Label, Pilot Study of the Safety and Clinical Effects of Rituximab in Patients with Rheumatoid Arthritis-Associated Interstitial Pneumonia. Open J. Rheumatol. Autoimmune Dis..

[27] Fui A., Bergantini L., Selvi E., Mazzei M.A., Bennett D., Pieroni M.G., Rottoli P., Bargagli E. (2020). Rituximab therapy in interstitial lung disease associated with rheumatoid arthritis. Intern. Med. J..

[28] Robles-Perez A., Dorca J., Castellví I., Nolla J.M., Molina-Molina M., Narváez J. (2020). Rituximab effect in severe progressive connective tissue disease-related lung disease: Preliminary data. Rheumatol. Int..

[29] Aletaha D., Neogi T., Silman A.J., Funovits J., Felson D.T., Bingham C.O., Birnbaum N.S., Burmester G.R., Bykerk V.P., Cohen M.D. (2010). 2010 Rheumatoid arthritis classification criteria: An American College of Rheumatology/European League Against Rheumatism collaborative initiative. Arthritis Rheum..

[30] Bohan A., Peter J.B. (1975). Polymyositis and dermatomyositis (first of two parts). N. Engl. J. Med..

[31] Bohan A., Peter J.B. (1975). Polymyositis and dermatomyositis (second of two parts). N. Engl. J. Med..

[32] Travis W.D., Costabel U., Hansell D.M., King T.E., Lynch D.A., Nicholson A.G., Ryerson C.J., Ryu J.H., Selman M., Wells A.U. (2013). An official American Thoracic Society/European Respiratory Society statement: Update of the international multidisciplinary classification of the idiopathic interstitial pneumonias. Am. J. Respir. Crit. Care Med..

[33] Moazedi-Fuerst F.C., Kielhauser S.M., Brickmann K., Hermann J., Lutfi A., Meilinger M., Brezinschek H.P., Graninger W.B. (2014). Rituximab for systemic sclerosis: Arrest of pulmonary disease progression in five cases. Results of a lower dosage and shorter interval regimen. Scand. J. Rheumatol..

[34] Wallace B., Vummidi D., Khanna D. (2016). Management of connective tissue diseases associated interstitial lung disease: A review of the published literature. Curr. Opin. Rheumatol..

[35] Nurmi H.M., Purokivi M.K., Kärkkäinen M.S., Kettunen H.P., Selander T.A., Kaarteenaho R.L. (2017). Are risk predicting models useful for estimating survival of patients with rheumatoid arthritis-associated interstitial lung disease?. BMC Pulm. Med..

[36] Xing N.S., Fan G.Z., Yan F., Liu Y.P., Zhang R. (2021). Safety and efficacy of rituximab in connective tissue disease-associated interstitial lung disease: A systematic review and meta-analysis. Int. Immunopharmacol..

[37] Hartung W., Maier J., Pfeifer M., Fleck M. (2012). Effective treatment of rheumatoid arthritis-associated interstitial lung disease by B-cell targeted therapy with rituximab. Case Rep. Immunol..

[38] Tashkin D.P., Roth M.D., Clements P.J., Furst D.E., Khanna D., Kleerup E.C., Goldin J., Arriola E., Volkmann E.R., Kafaja S. (2016). Mycophenolate mofetil versus oral cyclophosphamide in scleroderma-related interstitial lung disease (SLS II): A randomised controlled, double-blind, parallel group trial. Lancet Respir. Med..

[39] Liossis S.N., Bounas A., Andonopoulos A.P. (2006). Mycophenolate mofetil as first-line treatment improves clinically evident early scleroderma lung disease. Rheumatology (Oxford).

[40] Simeón-Aznar C.P., Fonollosa-Plá V., Tolosa-Vilella C., Selva-O’Callaghan A., Solans-Laqué R., Vilardell-Tarrés M. (2011). Effect of mycophenolate sodium in scleroderma-related interstitial lung disease. Clin. Rheumatol..

[41] Mendoza F.A., Nagle S.J., Lee J.B., Jimenez S.A. (2012). A prospective observational study of mycophenolate mofetil treatment in progressive diffuse cutaneous systemic sclerosis of recent onset. J. Rheumatol..

[42] Volkmann E.R., Tashkin D.P., Li N., Roth M.D., Khanna D., Hoffmann-Vold A.M., Kim G., Goldin J., Clements P.J., Furst D.E. (2017). Mycophenolate Mofetil Versus Placebo for Systemic Sclerosis-Related Interstitial Lung Disease: An Analysis of Scleroderma Lung Studies I and II. Arthritis Rheumatol..

[43] Naidu G.S.R.S.N.K., Sharma S.K., Adarsh M.B., Dhir V., Sinha A., Dhooria S., Jain S. (2020). Effect of mycophenolate mofetil (MMF) on systemic sclerosis-related interstitial lung disease with mildly impaired lung function: A double-blind, placebo-controlled, randomized trial. Rheumatol. Int..

[44] Fischer A., Brown K.K., Du Bois R.M., Frankel S.K., Cosgrove G.P., Fernandez-Perez E.R., Huie T.J., Krishnamoorthy M., Meehan R.T., Olson A.L. (2013). Mycophenolate mofetil improves lung function in connective tissue disease-associated interstitial lung disease. J. Rheumatol..

[45] Swigris J.J., Olson A.L., Fischer A., Lynch D.A., Cosgrove G.P., Frankel S.K., Meehan R.T., Brown K.K. (2006). Mycophenolate mofetil is safe, well tolerated, and preserves lung function in patients with connective tissue disease-related interstitial lung disease. Chest.

[46] Fraticelli P., Fischetti C., Salaffi F., Carotti M., Mattioli M., Pomponio G., Gabrielli A. (2018). Combination therapy with rituximab and mycophenolate mofetil in systemic sclerosis. A single-centre case series study. Clin. Exp. Rheumatol..

[47] Narváez J., LLuch J., Molina-Molina M., Vicens-Zygmunt V., Luburich P., Yañez M.A., Nolla J.M. (2020). Rituximab as a rescue treatment added on mycophenolate mofetil background therapy in progressive systemic sclerosis associated interstitial lung disease unresponsive to conventional immunosuppression. Semin. Arthritis Rheum..

[48] Bejan-Angoulvant T., Naccache J.M., Caille A., Borie R., Nunes H., Ferreira M., Cadranel J., Crestani B., Cottin V., Marchand-Adam S. (2020). Evaluation of efficacy and safety of rituximab in combination with mycophenolate mofetil in patients with nonspecific interstitial pneumonia non-responding to a first-line immunosuppressive treatment (EVER-ILD): A double-blind placebo-controlled randomized trial. Respir. Med. Res..

[49] Andersson H., Sem M., Lund M.B., Aaløkken T.M., Günther A., Walle-Hansen R., Garen T., Molberg Ø. (2015). Long-term experience with rituximab in anti-synthetase syndrome-related interstitial lung disease. Rheumatology (Oxford).

[50] Duarte A.C., Cordeiro A., Fernandes B.M., Bernardes M., Martins P., Cordeiro I., Santiago T., Seixas M.I., Ribeiro A.R., Santos M.J. (2019). Rituximab in connective tissue disease-associated interstitial lung disease. Clin. Rheumatol..

[51] Lee Y.H., Cha S.I., Lim J.K., Yoo S.S., Lee S.Y., Lee J., Kim C.H., Park J.Y. (2019). Clinical and radiological features of pulmonary tuberculosis in patients with idiopathic pulmonary fibrosis. Respir. Investig..

[52] Langlois V., Gillibert A., Uzunhan Y., Chabi M.L., Hachulla E., Landon-Cardinal O., Mariampillai K., Champtiaux N., Nunes H., Benveniste O. (2020). Rituximab and Cyclophosphamide in Antisynthetase Syndrome-related Interstitial Lung Disease: An Observational Retrospective Study. J. Rheumatol..

