# Supplementary material for: Efficacy and Safety of Rituximab in Autoimmune Disease—Associated Interstitial Lung Disease: A Prospective Cohort Study
Source: J Clin Med. 2022 Feb 10;11(4):927. doi: 10.3390/jcm11040927 (PMC8879100; doi:10.3390/jcm11040927)
Supplement: Supplementary file 1 [file jcm-11-00927-s001.zip › jcm-1569288-supplementary.pdf]

Supplementary Table S1: Previous treatment in 37 patients with CTD-ILD receiving rituximab.

Variable	Total *n*=37	RA *n*=19	SS *n*=14	IM *n*=4	*p* Value
csDMARD, *n* (%)	23 (62.0)	18 (94.7)	4 (28.6)	1 (25.0)	<0.001
Methotrexate, *n* (%)	20 (54.1)	17 (89.5)	3 (21.4)	0 (0.0)	<0.001
Leflunomide, *n* (%)	9 (24.3)	9 (47.4)	0 (0.0)	0 (0.0)	0.004
Sulfasalazine, *n* (%)	5 (13.5)	5 (26.3)	0 (0.0)	0 (0.0)	0.065
Hydroxychloroquine, *n* (%)	5 (13.5)	3 (15.8)	1 (7.1)	1 (25.0)	0.600
No. of previous csDMARDs, median (IQR)	1.0 (0.0-2.0)	2.0 (1.0-3.0)	0.0 (0.0-1.0)	0.0 (0.0-0.7)	<0.001
bDMARDs	11 (29.7)	11 (57.9)	0 (0.0)	0 (0.0)	0.001
Infliximab, *n* (%)	2 (5.4)	2 (10.5)	0 (0.0)	0 (0.0)	0.367
Etanercept, *n* (%)	4 (10.8)	4 (21.1)	0 (0.0)	0 (0.0)	0.120
Adalimumab, *n* (%)	3 (8.1)	3 (15.8)	0 (0.0)	0 (0.0)	0.213
Tocilizumab, *n* (%)	1 (2.7)	1 (5.3)	0 (0.0)	0 (0.0)	0.615
Abatacept, *n* (%)	1 (2.7)	1 (5.3)	0 (0.0)	0 (0.0)	0.615
No. of previous bDMARDs, median (IQR)	0.0 (0.0-1.0)	1.0 (0.0-2.0)	0.0 (0.0-0.0)	0.0 (0.0-0.0)	0.001
Immunosuppressants	16 (43.2)	7 (36.8)	8 (57.1)	1 (25.0)	0.375
Mycophenolate, *n* (%)	7 (18.9)	0 (0.0)	7 (50.0)	0 (0.0)	0.001
Azathioprine, *n* (%)	9 (24.3)	3 (15.8)	5 (35.7)	1 (25.0)	0.419
Cyclophosphamide, *n* (%)	8 (21.6)	4 (21.1)	4 (28.6)	0 (0.0)	0.471
No. of previous immunosuppressants, median (IQR)	1.0 (0.0-1.0)	0.0 (0.0-1.0)	1.0 (0.0-2.2)	0.0 (0.0-1.0)	0.033

Abbreviations: CTD: connective tissue disease; ILD: interstitial lung disease; RA: rheumatoid arthritis; IM: inflammatory myopathy; SS: systemic sclerosis; csDMARD: conventional synthetic disease-modifying antirheumatic drug; bDMARD: biologic DMARD; IQR: interquartile range. Statistical tests used: Pearson chi-squared ($\chi 2$), ANOVA, and Kruskal-Wallis.

Supplementary Table S2: Characteristics of patients with CTD-ILD receiving treatment with rituximab who died.

Patient	Type of CTD	Age (years)	Time since onset of ILD (months)	Treatment	Time receiving rituximab (months)	Cause of death
Patient 1	RA	73.1	93.2	Rituximab	71.8	Progression of ILD and pulmonary superinfection
Patient 2	RA	73.9	77.6	Rituximab and hydroxychloro-quine	23.4	Progression of ILD and pulmonary superinfection
Patient 3	RA	64.2	81.0	Rituximab and sulfasalazine	74.1	Progression of ILD and pulmonary superinfection
Patient 4	RA	78.5	85.8	Rituximab and mycophenolate	12.0	Progression of ILD
Patient 5	RA	76.5	57.2	Rituximab	60.1	Progression of ILD and pulmonary superinfection
Patient 6	RA	53.3	124.6	Rituximab and hydroxychloro-quine	80.2	Progression of ILD
Patient 7	SS	58.1	28.5	Rituximab and methotrexate	24.0	Progression of ILD

Abbreviations: SAD: systemic autoimmune disease; ILD: interstitial lung disease; RA: rheumatoid arthritis; SS: systemic sclerosis.

Supplementary Table S3: Pulmonary function results at ILD diagnosis and at baseline in patients with CTD-ILD.

Variable		Total *n*=37	RA *n*=19	SS *n*=14	IM *n*=4	*p* Value
Pulmonary function tests						
FVC, mean (SD)	ILD-diagnosis	73.5 (16.9)	73.0 (13.1)	73.9(21.7)	82.7 (14.5)	0.485
	Baseline	72.2 (21.3)*	69.1 (15.0)*	71.6(21.7)*	79.0 (15.0)*	0.644
FEV1, mean (SD)	ILD-diagnosis	76.1 (18.1)	72.0 (16.9)	79.7 (21.7)	85.0 (3.5)	0.332
	Baseline	73.0 (18.8)*	69.8 (16.0)*	76.9 (24.9)*	76.2 (7.5)*	0.570
DLCO-SB, mean (SD)	ILD-diagnosis	58.3 (16.1)	58.1 (18.4)	54.8 (15.6)	61.2 (7.0)	0.890
	Baseline	55.9 (15.7)*	56.2 (17.7)*	52.8 (15.6)*	58.0 (5.0)*	0.935

Abbreviations: CTD: connective tissue disease; ILD: interstitial lung disease; RA: rheumatoid arthritis; IM: inflammatory myopathy; SS: systemic sclerosis; FVC: forced vital capacity; FEV1: forced expiratory volume in the first second; DLCO: diffusing capacity of the lungs for carbon dioxide; *$p<0.005$ ILD diagnosis vs baseline; Statistical tests used: Pearson chi-squared ($\chi 2$), ANOVA, Kruskal-Wallis, paired t test, and Wilcoxon test.

Supplementary Table S4: Factors associated with pulmonary outcomes in patients with CTD-ILD receiving rituximab.

Variable	Improvement stabilization (*n*=23)	Progression-death (*n*=14)	*p* Value
Epidemiological characteristics			
Sex, female, *n* (%)	16 (69.6)	11 (78.6)	0.550
Age in years, mean (SD)	61.2 (9.9)	65.4 (9.6)	0.224
Race, Caucasian, *n* (%)	23 (100.0)	13 (92.9)	0.194
Clinical-analytical characteristics			
Smoking			0.286
Never smoked, *n* (%)	14 (60.9)	6 (42.9)	
Smoked, *n* (%)	9 (39.1)	8 (57.1)	
Duration of CTD, months, median (IQR)	79.0 (41.6-180.0)	130.9 (87.3-200.8)	0.139
Duration of ILD, months, median (IQR)	54.9 (26.0-93.4)	86.5 (51.9-130.8)	**0.046**
RF-positive (>10) *n* (%)	10 (43.5)	9 (64.3)	0.219
ACPA (<20), *n* (%)	9 (39.1)	9 (64.3)	0.138
ANA-positive, *n* (%)	16 (69.6)	8 (57.1)	0.443
Anti-scl70, *n* (%)	4 (17.4)	3 (21.4)	0.761
Anticentromere, *n* (%)	4 (17.4)	0 (0.0)	0.098
Anti-RNA polymerase 3, *n* (%)	1 (4.3)	0 (0.0)	0.429
Anti Ku, *n* (%)	1 (4.3)	0 (0.0)	0.429
Anti-PL7, *n* (%)	2 (8.7)	0 (0.0)	0.257
Anti-EJ, *n* (%)	1 (4.3)	0 (0.0)	0.429
Anti-TIF, *n* (%)	1 (4.3)	0 (0.0)	0.429
Radiological pattern			0.284
UIP, *n* (%)	9 (39.1)	9 (64.3)	
NSIP, *n* (%)	13 (56.5)	5 (35.7)	
Fibrotic NSIP, *n* (%)	1 (4.3)	0 (0.0)	
Baseline pulmonary function tests			
FVC, mean (SD)	75.5 (19.0)	61.7 (14.7)	**0.045**
FVC<80%, *n* (%)	12 (52.2)	12 (85.7)	**0.038**
FEV_1, mean (SD)	75.9 (18.5)	69.1 (19.6)	0.248
DLCO-SB, mean (SD)	59.8 (16.0)	49.5 (13.3)	**0.036**
Treatment			
Time to initiation of RTX*, median (IQR)	7.4 (6.0-22.7)	42.4 (14.2-84.6)	**0.016**
Time with RTX, median (IQR)**	38.2 (24.0-63.3)	42.4 (17.0-72.3)	0.842
Combination with DMARDs, *n* (%)	9 (39.1)	6 (42.9)	0.823
Methotrexate, *n* (%)	3 (13.0)	2 (14.3)	0.915
Leflunomide, *n* (%)	2 (8.7)	0 (0.0)	0.257
Sulfasalazine, *n* (%)	0 (0.0)	1 (7.1)	0.194
Hydroxychloroquine, *n* (%)	4 (17.4)	3 (21.4)	0.761
Combination with immunosuppressants, *n* (%)	15 (65.2)	5 (35.7)	0.081
Mycophenolate, *n* (%)	15 (65.2)	4 (28.6)	**0.031**
Azathioprine, *n* (%)	0 (0.0)	1 (7.1)	0.194
Corticosteroids, *n* (%)	16 (69.6)	9 (64.3)	0.739
Dose of corticosteroids, median (IQR)	5.0 (0.0-10.0)	7.5 (5.0-10.0))	0.476

Abbreviations: CTD: connective tissue disease; ILD: interstitial lung disease; RTX: rituximab; ACPA: anticitrullinated peptide antibody; ANA: antinuclear antibody; FVC: forced vital capacity; FEV1: forced expiratory volume in the first second; DLCO: diffusing capacity of the lungs for carbon monoxide; UIP: usual interstitial pneumonia; NSIP: nonspecific interstitial pneumonia; HRCT: high-resolution computed tomography; csDMARD: conventional systemic disease-modifying antirheumatic drug. * Time from diagnosis of ILD to initiation of treatment with RTX ** Time since initiation of treatment with RTX to end of follow-up or death. Statistical tests used: Pearson chi-squared ($\chi 2$), ANOVA, and Kruskal-Wallis.
